# Pinniped- and Cetacean-Derived ETosis Contributes to Combating Emerging Apicomplexan Parasites (*Toxoplasma gondii*, *Neospora caninum*) Circulating in Marine Environments

**DOI:** 10.3390/biology8010012

**Published:** 2019-03-09

**Authors:** Rodolfo Villagra-Blanco, Liliana M. R. Silva, Iván Conejeros, Anja Taubert, Carlos Hermosilla

**Affiliations:** Institute of Parasitology, Justus Liebig University Giessen, 35392 Giessen, Germany; Liliana.Silva@vetmed.uni-giessen.de (L.M.R.S.); Ivan.Conejeros@vetmed.uni-giessen.de (I.C.); Anja.Taubert@vetmed.uni-giessen.de (A.T.); Carlos.R.Hermosilla@vetmed.uni-giessen.de (C.H.)

**Keywords:** extracellular traps, ETosis, emerging marine diseases, marine mammals, neozoan parasites

## Abstract

Leukocytes play a major role in combating infections either by phagocytosis, release of antimicrobial granules, or extracellular trap (ET) formation. ET formation is preceded by a certain leukocyte cell death form, known as ETosis, an evolutionarily conserved mechanism of the innate immune system also observed in marine mammals. Besides several biomolecules and microbial stimuli, marine mammal ETosis is also trigged by various terrestrial protozoa and metazoa, considered nowadays as neozoan parasites, which are circulating in oceans worldwide and causing critical emerging marine diseases. Recent studies demonstrated that pinniped- and cetacean-derived polymorphonuclear neutrophils (PMNs) and monocytes are able to form different phenotypes of ET structures composed of nuclear DNA, histones, and cytoplasmic peptides/proteases against terrestrial apicomplexan parasites, e.g., *Toxoplasma gondii* and *Neospora caninum*. Detailed molecular analyses and functional studies proved that marine mammal PMNs and monocytes cast ETs in a similar way as terrestrial mammals, entrapping and immobilizing *T. gondii* and *N. caninum* tachyzoites. Pinniped- and cetacean leukocytes induce vital and suicidal ETosis, with highly reliant actions of nicotinamide adenine dinucleotide phosphate oxidase (NOX), generation of reactive oxygen species (ROS), and combined mechanisms of myeloperoxidase (MPO), neutrophil elastase (NE), and DNA citrullination via peptidylarginine deiminase IV (PAD4).This scoping review intends to summarize the knowledge on emerging protozoans in the marine environment and secondly to review limited data about ETosis mechanisms in marine mammalian species.

## 1. Introduction

Early innate immune responses are important mechanisms of the host defense against infections, either protecting non-vertebrate organisms or synergizing adaptive immunity in vertebrate animals [1,2]. These responses are performed by anatomical and physiological barriers (e.g., mucociliary blanket), antimicrobial factors (e.g., complement, lysozyme, lactoferrin, defensins and reactive oxygen and nitrogen intermediates), and by professional mononuclear phagocytes (e.g., polymorphonuclear neutrophils (PMNs), monocytes, macrophages), all of them representing the first line of defense against a vast number of potentially pathogenic agents [3]. These leukocytes are able to entrap, phagocytize, and damage invasive microorganisms, but at the same time they release cytokines/chemokines [4,5,6,7], which are responsible for further leukocyte recruitment, starting acute systematic immune responses and inflammation processes [8,9,10,11].

Marine mammals, especially cetaceans (whales and dolphins), sirenians (manatees and dugongs), pinnipeds (seals, sea lions, sea leopards and walruses), polar bears, and sea otters are the only mammalian species which spend all or majority of their lives in marine environments. Marine mammals can not only be found in the open ocean, but also in freshwaters of lakes [12] and rivers [13], thereby diversifying the potential contact with humans, domestic animals and anthropozoonotic pathogens [14].

Further, marine mammals are highly valuable with respect to comparative and evolutionary immunology since they are the only descendants of primitive terrestrial mammals which returned to the sea and their immune system was initially adapted to a terrestrial existence, including host–parasite interactions that have been re-evolved in aquatic ecosystems [15]. Several studies have described the mechanisms involved in cetacean and pinniped innate immune system [9,10,11,12,13,14,15,16,17,18,19,20,21,22,23,24,25], highlighting the importance of leukocytes as forefront of defense against infections [26,27], which is highly important for inflammation resolution and/or wound healing [28]. Moreover, anthropogenic immunosuppression can seriously affect leukocyte counts and generate differences in immune reactions between individuals from the same marine group, e.g., in sirenians, sea otters, pinnipeds, and cetaceans, thereby causing mass mortalities of classical terrestrial pathogens, such as *Morbillivirus* and *Toxoplasma gondii* in marine ecosystems worldwide [11,29,30,31,32,33].

Therefore, this review focuses on both, oceanic emerging neozoan parasites and on the innate immune system of pinniped/cetacean mammals. It additionally reviews the innate effector defense mechanism of ETosis and summarizes very limited data on *T. gondii-* and *Neospora caninum*-induced ETosis in pinnipeds and cetaceans, thereby highlighting the relevance of this ancient, conserved and effective defense mechanism against these parasites currently circulating in marine environments [31,32,33,34,35,36,37].

## 2. Marine Environment Affected by Emerging Neozoan Parasites

Oceans are currently threatened by the presence of opportunistic emerging neozoan pathogens (i.e., viruses, bacteria, fungi and parasites) affecting both animal and human health and welfare [36,37]. Typical terrestrial neozoan parasites, such as apicomplexan parasites *T. gondii, N. caninum, Sarcocystis canis*, and *Cryptosporidium* spp. (i.e., *C. parvum, C. hominis)* as well as enteropathogenic protozoans, like *Giardia intestinalis, Balantidium* spp., and *Entamoeba* spp., have been reported in wild populations of diverse marine mammals [38,39,40,41,42,43,44] and in different marine ecosystems in past decades [13,42,43,44,45,46,47,48,49], causing lethal infections not only in sea otters [31,32] and large whales [36,49,50,51,52], but also in pinnipeds [53] and dolphins [34,42].

Occurrence of coccidian parasitoses such as toxoplasmosis, neosporosis, and sarcocystosis are quite problematic in marine mammals reflecting contamination status of oceans and coastal waters with infectious sporulated oocysts [31,34,35,54]. As recorded for terrestrial mammalians, congenital toxoplasmosis has also been reported in cetaceans, such as the Indo-Pacific bottlenose dolphin (*Tursiops aduncus*) [55]. Additionally, Dubey et al. [56] showed *T. gondii* tissue cyst formation in striped dolphins (*Stenella coeruleoalba*). Obviously, a high contamination of aquatic ecosystems with infectious oocysts of *T. gondii-, N. caninum-, S. canis-,* and *C. parvum*, and cysts of *G. intestinalis*, *Entamoeba* spp., or *Balantidium* spp. will facilitate infections in wild marine mammals [31,37,56]. Moreover, movements and migration of uninfected marine mammals into areas with endemic oocyst/cyst contamination prompted by environmental changes, such as “El Niño” events and/or global warming, might result in disease outbreaks as demonstrated for terrestrial mammals [57]. Additionally, direct and indirect contacts between humans and marine mammals are nowadays more frequent, especially due to urbanization expansion along the coasts, tourist activities like whale- and dolphin-watching, aquatic sport activities, rehabilitation and research practices involving maintenance of sick or injured marine mammals, and the contact of marine mammals with pathogens from domestic pets and livestock [58].

To date, a large number of parasite species have gained importance as opportunistic neozoan infections in the marine environment [36,47,49,56,57,58,59,60,61,62,63,64,65,66]. Particularly, *T. gondii* and *Sarcocystis neurona* stages in brain tissue were associated with encephalitis in stranded harbor seals [65,66]. Consequently, specific antibodies against the apicomplexan parasites, such as *N. caninum, T. gondii,* and *S. neurona*, were recently reported in dolphins [50,55,63,64,65,66,67,68,69], whales [51,70], sea otters [31,71] and seals [44,59] confirming a rather wide ocean contamination. Whilst the contamination of oceanic environment and coastal waters with sporulated oocysts and infective cysts is quite obvious [31,41], molecular mechanisms of host innate and adaptive immune responses of marine mammals against these neozoans are still unclear.

## 3. Cetacean/Pinniped Leukocytes of the Innate Immune System

The innate immune system is one of the two main branches of host defense in vertebrates, with the other one being the adaptive immune system. The innate immune system is evolutionary older and is known as the dominant immune system, acting as a first line of defense against invasive pathogens [26]. The innate immune system of mammals is mainly composed of professional phagocytes (i.e., PMNs, monocytes and macrophages) and highly immunoreactive host epithelial- and host endothelial cells covering mucosal and vessel surfaces of the body.

The innate immune system of cetaceans/pinnipeds mainly includes PMNs and monocytes, which represent 22–72% and 0–11% of the circulating leukocytes, respectively [38,72,73,74,75,76]. As seen in terrestrial mammals, also eosinophils, mast cells, basophils, and macrophages are found in marine mammals [39,76]. Leukocyte morphology in healthy marine and terrestrial mammals are quite similar, but with some notable exceptions [73,74,75,76,77,78,79]. PMNs of most marine mammals have round to oval granules that either do not stain or stain as pale pink within heterophilic granulocytes using Wright-Giemsa [75,77]. These heterophilic granules contain myeloperoxidase (MPO) and neutrophil elastase (NE), which are also typical terrestrial mammalian PMN-derived antimicrobial proteins, identified in PMNs of humans, horses, cattle, goats, and dogs [80,81,82,83,84].

Interestingly, cetaceans have much higher eosinophil counts than most other mammals, including pinnipeds [76,79]. Eosinophils in tissues react to chemoattractants generated in response to parasites, as recently reported for other terrestrial mammalian species [47,85]. They release eosinophil-derived peroxidase from their granules and interact by hydrogen peroxide to perform respiratory burst activities with halide ions or via extracellular trap (ET) formation [85,86]. This complex, combined with other oxygen species and the major basic protein (MBP) being released from secondary granules, enables eosinophils to display bactericidal and parasiticidal activities [85,87,88]. Marine mammal effector mechanisms of leukocytes, such as phagocytosis and respiratory burst resulting in reactive oxygen species (ROS) production, have been well investigated [13,14,15,16,17,18,22,89] and show similar pathogen killing capacities as leukocytes of terrestrial mammals [90,91,92,93,94].

As described for terrestrial mammalians, age-related variations in leukocyte composition have also been recorded for marine mammals. As such, Hasselmeier et al. [93] reported that more than half of a free-living harbor seal (*Phoca vitulina*) pup population in the Northern Sea showed a proportion of at least 10% monocytes in the blood during the winter, while yearlings totally lacked this cell type during the spring season. Differences in numbers, activation status, and monocyte-mediated phagocytosis were most probably due to diverse stress pressure and/or concomitant indiscernible infections [93].

## 4. ETosis in Terrestrial and Marine Mammals

The paradigm of how mammalian PMNs combat, entrap, and kill pathogenic agents has deeply been changed after the landmark publication of Brinkmann et al. [80]. The discovery of DNA-based antimicrobial ETs not only revolutionized our knowledge on early host innate immune reactions, but changed understanding of their functions in metabolic, autoimmune, reproductive, thromboimmune, tumoral, and inflammatory disorders [94,95,96,97,98,99]. ET-related DNA-structures have widely been studied in context of diverse PMN-derived antibacterial effector defense mechanisms, and were originally named as neutrophil extracellular traps (NETs). Meanwhile, other types of professional phagocytes, such as mast cells [100,101,102], eosinophils [103], macrophages [104,105,106,107], basophils [108,109], and monocytes [110,111,112,113],were also described as capable for ET extrusion. The process of leukocyte-mediated ET release into extracellular space is known in literature as ETosis [80,114].

Mammalian ETosis can be induced by a variety of potent stimulators, including soluble molecules (e.g., granulocyte-macrophage colony-stimulating factor (GM-CSF)/complement factor 5a [114,115], activated platelets [116], toll-like receptors (TLRs) [117], interleukin 8 (IL-8), interferon gamma (IFNγ) [118], lipopolysaccharides (LPS) [80], phorbol 12-myristate 13-acetate (PMA) [119,120], zymosan [38,82], singlet oxygen [121], fragment crystallizable receptor (Fc receptor) [122,123,124], mycotoxins [125],and invasive pathogens (e.g., bacteria [80,104,126], fungi [122,123,127], viruses [128,129], and parasites [27,111,130,131,132]).

**Figure 1:**
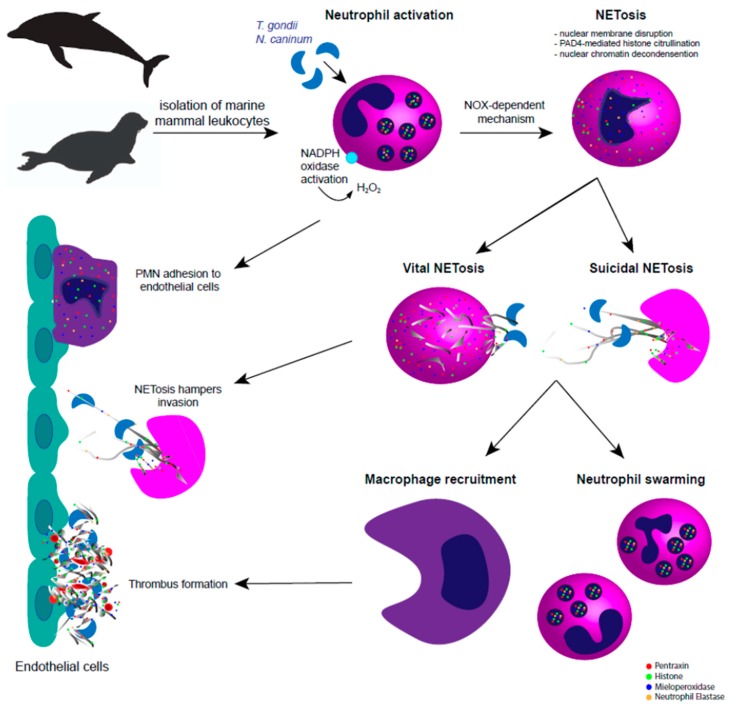
Apicomplexan-triggered neutrophil extracellular traps (NETosis) in pinniped and cetacean species. In the pinniped and cetacean system, tachyzoite-derived extracellular traps (ETosis) is dependent on nicotinamide adenine dinucleotide phosphate oxidase (NOX) pathways, via reactive oxygen species (ROS) activation and histone citrullination via peptidylarginine deiminase IV (PAD4). Marine mammal-triggered vital and suicidal ETosis result in effective entrapment of *Toxoplasma gondii* and *Neospora caninum* tachyzoites, thereby hampering active invasion of host endothelial cells. After extracellular trap (ET) release, complementary immune mechanisms take place to keep the homeostasis and to hamper ETosis-mediated collateral tissue damage, such as macrophage recruitment, polymorphonuclear neutrophil (PMN) swarming, chemotaxis, activation of endothelium, and immunothrombosis.

Efficient mammalian ETosis requires mature leukocytes with physiological activities of reactive oxygen species (ROS), nicotinamide adenine dinucleotide phosphate oxidase (NOX), neutrophil elastase (NE), myeloperoxidase (MPO) and peptidylarginine deiminase type IV (PAD4) [83,133,134,135,136,137]. Upon respective stimulation of leukocytes, the nuclear envelope disintegrates, thereby permitting the mixture of chromatin with granular proteins/peptides [80]. NE and MPO degrade nuclear histones (H1, H2A/H2B, H3, H4) and stimulate chromatin decondensation [134] through hypercitrullination of specific histones via PAD4, which allows electrostatic coiling of chromatin [135,138]. DNA complexes being decorated with granular proteins/peptides and nuclear histones are consequently extruded from dying cells in fine structures to the extracellular environment (Figure 1). This mechanism is known as suicidal ETosis [139] and lytic ETosis was found associated with other ROS-related activation mechanisms, which have also been observed in leukocytes of marine mammals [140]. Moreover, mammalian ETosis requires the activation of intracellular signaling pathways, frequently involving mitogen-activated protein kinases (MAPK), such as Raf-MEK-ERK kinases as well as p38 MAPK routes [136,137,141]. Additionally, ETosis revealed as a calcium-dependent process in various vertebrate species [137,142,143,144].

As stated above, suicidal ETosis is mainly known as NOX-dependent cell death pathway [26,82,111,136,137], however, NOX-independent suicidal ETosis has been also reported [121,145,146]. NOX-independent suicidal ETosis includes a considerable lower activity of extracellular signal-regulated kinases (ERK/MAPK) and rather moderate levels of protein kinase B (PKB or AKT) activation, i.e., of molecules known to be important regulators of autophagy and oncogenic processes. Meanwhile, the activation of p38 appears similar in both, lytic and non-lytic ETosis [126,144,145,147]. Interestingly, even singlet oxygen can stimulate mammalian ETosis in a NOX-independent manner [121]. Irrespective of NOX-dependency, parasites may either be immobilized or entrapped within sticky DNA fibers [27] or be killed via the local high concentration of effector molecules [130].

Since the description of Malawista et al. [148] in which enucleated PMNs survived after being confronted with invasive pathogens despite their short lifespan, posterior studies confirmed these findings proving that some leukocytes do not necessarily succumb during ETosis [103,126,149]. In this context, Yousefi et al. [103] demonstrated that eosinophils and certain PMN subpopulations release ETs of mitochondrial sources without losing their vitality, known as non-lytic or vital ETosis. Consequently, Yipp et al. [150] verified that PMNs which performed vital NETosis were still viable and retained their capability to engulf bacteria via phagocytosis. However, the exact routes of vital ETosis through NOX-independent mechanisms and the release of mitochondrial DNA are not clear yet. Interestingly, NOX-independent vital ETosis seems faster than NOX-dependent suicidal ETosis and seem to rely on a vesicular-based pathway leading to nuclear DNA release, known as vesicular ETosis [97,126,145].

Furthermore, in vitro and in vivo release of different phenotypes of ETs depends on stimulus and probably simultaneously involves several molecular pathways. Recently, three different phenotypes of ETs have been described in PMNs and eosinophils, e.g., the so-called aggregated (*agg*ETs), spread (*spr*ETs), and diffuse (*diff*ETs) ETs [83,151]. Consistent with these findings, *agg*ETs, *spr*ETs, and *diff*ETs have been also recently observed in pinnipeds and dolphins [38,39,40].

While a vast amount of investigations have been performed unveiling precise cellular processes occurring during suicidal, vital and vesicular ETosis, many aspects concerning receptors and signaling pathways being involved in these effector mechanisms are still unresolved. The same holds true for studies on pinniped/cetacean-derived ETosis.

**Figure 2:**
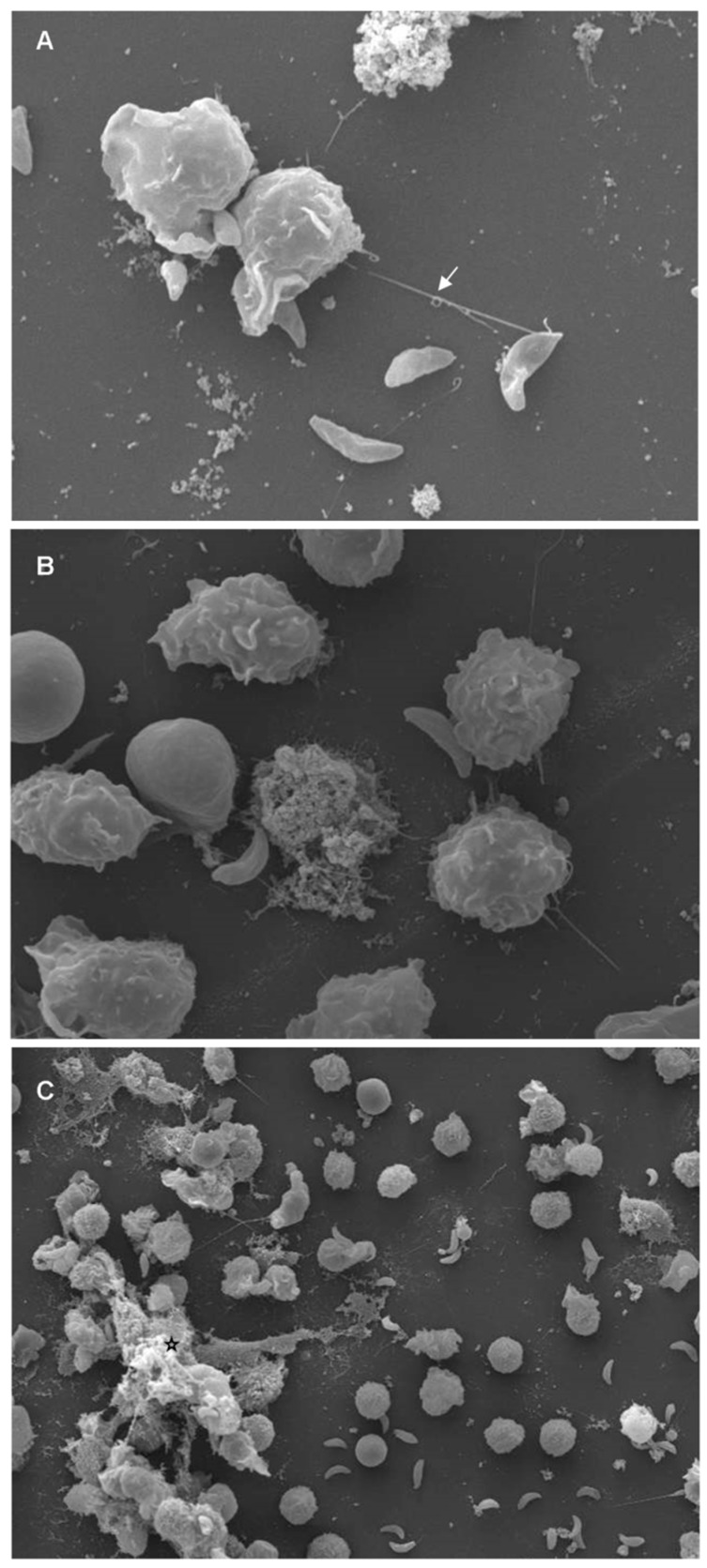
*Toxoplasma gondii*-triggered cetacean extracellular traps (ETosis) visualized by scanning electron microscopy (SEM) analysis. Polymorphonuclear neutrophils (PMN) of bottlenose dolphins (*Tursiops truncatus*) were isolated from whole blood and confronted with viable *T. gondii* tachyzoites (ratio 1:4) for 60 min on poly-L-lysine (Sigma Aldrich)-pretreated 10 mm coverslips (10 mm of diameter; Nunc) (Please see Supplementary Materials). Samples were fixed in a medium containing formaldehyde 2% (Merck) and 2.5 % glutaraldehyde (Merck), post-fixed in 1 % osmium tetroxide, washed with distilled water before dehydration and critical point dry with CO_2_-application, and sprinkled with gold particles. The specimens were examined using scanning electron microscope (Philips ^®^ XL30, Philips, Amsterdam, The Netherlands). (**a**) Vital cetacean PMNs extruded a neutrophil extracellular trap (NET)-like delicate filament (white arrow), attached to a *T. gondii*-tachyzoite. (**b**) Many activated PMNs changed their habitual round morphology after they entered in contact with tachyzoites. **(c)** Conglomerate of parasites being entrapped in a rather thicker DNA meshwork of cetacean-PMN-released fibers (black star).

In line with ultrastructural scanning electron microscopy (SEM) findings on terrestrial mammals, marine mammal-derived suicidal ETosis was also described in response to neozoan *T. gondii* and *N. caninum* (Figure 2 and Supplementary Materials). These data demonstrate that this cell death process is most probably an early, well-conserved and ancient host innate defense mechanism. Prior to suicidal ETosis, dolphin-and pinniped-derived PMNs and monocytes firmly attached to and subsequently entrapped *N. caninum/T. gondii* tachyzoites by releasing sticky ETs [38,39]. Thereby active tachyzoite host cell invasion was impeded, as already shown for other apicomplexans in vitro and in vivo [32,85,111,137]. Considering that *T. gondii* and *N. caninum* are obligate intracellular parasites and rely on host cell metabolism modulation for successful proliferation [152,153,154,155,156], the blockage of tachyzoite-derived host cell invasion via suicidal ETosis seems to be a crucial antiparasitic effect. Thus, ETosis-mediated inhibition host cell invasion might indeed exhibit a detrimental impact on parasitic development and outcome of disease as postulated elsewhere (for reviews refer to [27,47]). As already demonstrated for other terrestrial mammals, such as dogs [83,157], cattle [158], goats [82,159,160], and sheep [161], the DNA nature of ETs was also confirmed for pinniped- and cetacean-derived leukocytes since treatments with deoxyribonuclease I (DNase I) resulted in a significant reduction of *T. gondii* and *N. caninum-*triggered ETosis [38,39].

It seems obvious that ETosis might be of relevance in marine mammals since the persistence of *T. gondii* in marine environments is nowadays growing through increased ocean contamination, by the facultative life cycle of this parasite, by a wide spectrum of suitable hosts and additional vertical transmission routes. Thus, an expansion of land-to-sea parasite colonization leading to frequent infections in marine mammals, such as sea otters or pinnipeds [162], will probably occur in future. The same might be true for the closely related euryxenous *N. caninum*, which shares similar aspects of biology and pathogenicity with *T. gondii* [39].

Interestingly, previous reports on NOX-derived intracellular ROS and dose-dependent ETosis triggered by in human [163] and bovine [111] PMNs were also confirmed for pinniped- and cetacean-triggered suicidal ETosis [38,39]. These observations might be associated with the pathogenicity of toxoplasmosis and neosporosis in marine mammals, since overwhelming suicidal ETosis may harm host tissues by different mechanisms, but especially through release of MPO, histones, chromatin, pro-inflammatory peptides/proteases, and oxidants, which, in turn, might trigger endothelial activation, apoptosis or necrosis in affected tissues or organs [164,165]. In contrast to parasite-induced NETosis in terrestrial mammals, pinniped-derived ETosis triggered by *T. gondii* revealed to be time-dependent [38]. However, *N. caninum*-induced ETosis in dolphins revealed as time-independent [39]. Observed differences might rely on the activation status of PMNs and final break-up of the cell membrane, i.e., each marine mammal species might require different time spans to fulfill the NETotic cascade [113], which is probably related with duration of vital and/or lethal ETosis as already demonstrated for other non-marine mammalian host species [147,150,166].

Inhibition of NOX-activity in pinniped- and cetacean-derived phagocytes via diphenylene iodondium (DPI) pre-treatments resulted in a significant diminishment of suicidal ETosis, thereby indicating NOX-dependency of this process. Accordingly, marine mammalian PMNs and monocytes appear to be capable of NOX activation, ROS production, and of performing ROS-dependent phagocytic activities [17,23,93,167]. Moreover, harbor seal (*P. vitulina*)-derived PMNs/monocytes and dolphin (*Tursiops truncatus*)-derived PMNs were proven to rapidly undergo lethal ETosis (within 10 min of exposure) against vital *T. gondii-*tachyzoites [38,40]. Thus, these cell types represent one of the fastest acting immune cell type undergoing ETosis, so far.

In line with pinniped/cetacean immune system, mammalian ETosis being performed in response to *Eimeria bovis, C. parvum,* and *Besnoitia besnoiti* stages is also known as a NOX-dependent mechanism [26,136,137,158,168], which finally leads to extrusion of nuclear and cytoplasmic granule enzymes, and the formation of DNA-enriched fibers being adorned with histones and granular proteins. For the latter, a variety of molecules was meanwhile identified to be present in ETosis, including NE, MPO, pentraxin, lactoferrin, cathepsin G, α-defensin, azurocidin, lysozyme, cathelicidins (LL-37), bacterial permeability-increasing protein (BPI), peptidoglycan recognition proteins (PGRPs), and other PMN granular components (for reviews see [26,27,47,169]). Whether these molecules are also found in marine mammal-triggered ETosis needs further investigations to be elucidated. Furthermore, it remains unclear whether NOX-independent suicidal ETosis is also performed by pinniped/cetacean leukocytes. The same holds true for any receptors (e.g., TLRs, CD11b) or ligands to be involved in apicomplexan-mediated ETosis [136].

## 5. Conclusions

Despite a vast number of investigations on terrestrial mammalian-triggered ETosis, it still remains enigmatic in pinniped/cetacean species, and various aspects of their nature and significance in vivo remain to be elucidated. Thus, specific signaling pathways or granular molecules leading to parasite entrapment by marine mammal-mediated ETosis are still unclear. The molecular composition of marine mammal-derived ETosis showed the presence of antimicrobial NE, MPO, and global histones, but their antimicrobial/antiparasitic role in vivo is actually unsolved. The actual role of different phenotypes of ETs in marine mammals in vivo also requests further detailed studies, in particular with respect to the impact of pro-inflammatory antimicrobial components, accumulating in high concentrations in organs and tissues in the case of *agg*ETs. In the same way, further studies are required concerning the different molecular activation mechanisms involved in ETosis, not only those ones associated with the leukocyte type involved in this process, but also the intraspecific differences between the diverse pathogenic agents and even between the marine mammals should be considered.

As also true for terrestrial mammalian-derived ETosis, the question why only a proportion of activated PMNs/monocytes are undergoing ETosis in response to parasites still has to be answered. Furthermore, the involvement of ETosis in several diseases (e.g., metabolic-, autoimmune-, reproductive-, tumor- and coagulopathy-related disorders) is another enigmatic field to be considered in marine mammals. In conclusion, we call for more detailed investigations in this fascinating field to better understand molecular aspects underlying degradation and regulation of marine mammal-derived ETosis. This investigation is of paramount importance given that ETosis releases vast amount of potent pro-inflammatory molecules into the system, which might have detrimental (in case of uncontrolled release) but also beneficial effects for the host by entrapping and inhibiting dissemination of parasites.

## Supplementary Materials

The following are available online at https://www.mdpi.com/2079-7737/8/1/12/s1, Supplementary Material: Scanning electron microscopy—Materials and methods applied for figure 2.
