# Supplementary material for: Pinniped- and Cetacean-Derived ETosis Contributes to Combating Emerging Apicomplexan Parasites (Toxoplasma gondii, Neospora caninum) Circulating in Marine Environments
Source: Biology (Basel). 2019 Mar 9;8(1):12. doi: 10.3390/biology8010012 (PMC6466332; doi:10.3390/biology8010012)
Supplement: Supplementary file 1 [file biology-08-00012-s001.pdf]

Supplementary Data

Scanning electron microscopy - Materials and methods applied for figure 2

Vital *Toxoplasma gondii*-tachyzoites were collected in supernatants of infected African green monkey kidney epithelial cell (MARC 145) monolayer, filtered with 5 ųm sterile syringe filters (Sartorius AG) to removed cell debris, washed thrice with sterile PBS (400 × *g*, 12 min), counted using a Neubauer haemocytometer chamber (Marienfeld) and re-suspended in sterile RPMI 1640 medium without phenol red (Gibco) until further experimental use. Cetacean PMN were co-cultured with these vital tachyzoites (ratio: 1:3) for 60 min on poly-L-lysine (Sigma-Aldrich) pre-coated coverslips (10 mm of diameter; Nunc). Cells were fixed in 2.5% glutaraldehyde (Merck), post-fixed in 1% osmium tetroxide (Merck), washed in distilled water, dehydrated, critical point dried by CO_2-treatment and sputtered with gold particles. Specimens were examined using a Philips XL30® scanning electron microscopy.
